# Supplementary material for: Vaccination Schedule and Age Influence Impaired Responsiveness to Hepatitis B Vaccination: A Randomized Trial in Central Asia
Source: Pathogens. 2024 Dec 9;13(12):1082. doi: 10.3390/pathogens13121082 (PMC11728755; doi:10.3390/pathogens13121082)
Supplement: Supplementary file 1 [file pathogens-13-01082-s001.zip › Figure S1.pdf]

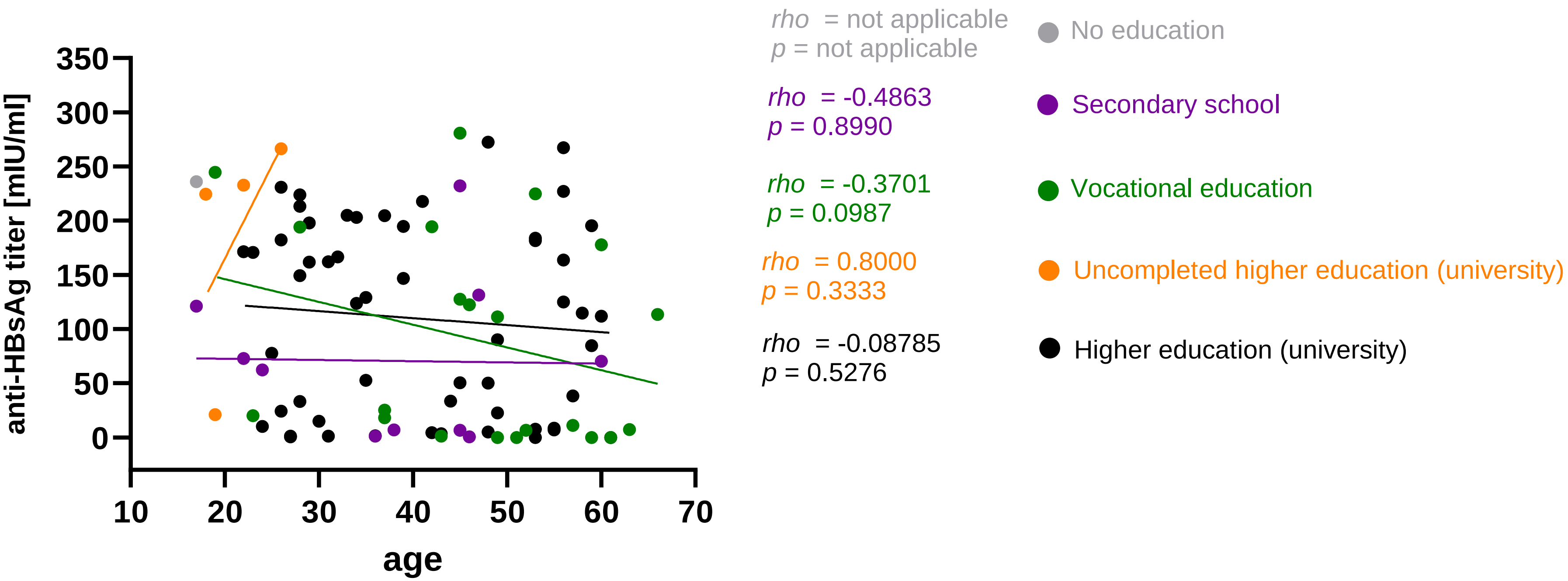

Supplementary Figure S1. Correlation analysis of age and education with anti-HBsAg titers after the 3rd vaccination. Associations between the age and education of study participants with antibody titers after the 3rd vaccination were done with the Spearman correlation test. No education (grey). Secondary school (purple). Vocational education (green). Uncompleted higher education (university) (orange). Higher education (university) (black).
