# Supplementary material for: Vaccination Schedule and Age Influence Impaired Responsiveness to Hepatitis B Vaccination: A Randomized Trial in Central Asia
Source: Pathogens. 2024 Dec 9;13(12):1082. doi: 10.3390/pathogens13121082 (PMC11728755; doi:10.3390/pathogens13121082)
Supplement: Supplementary file 1 [file pathogens-13-01082-s001.zip › Figure S2.pdf]

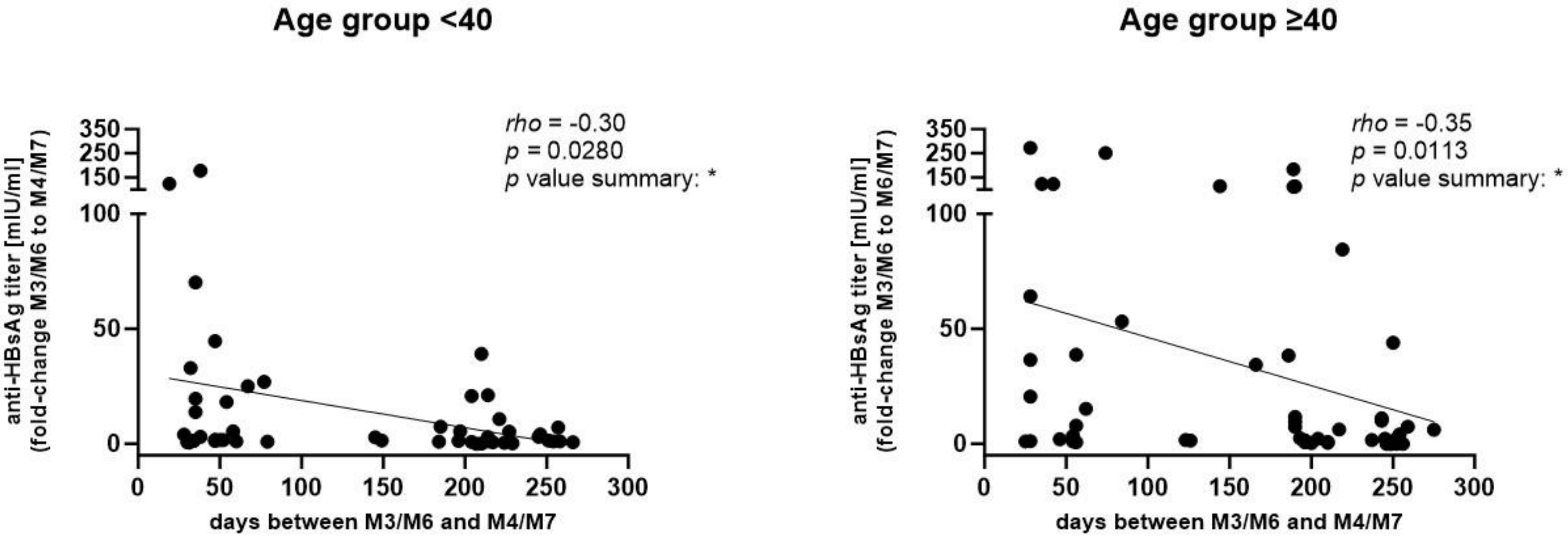

Supplementary Figure S2. Correlation analysis of change in anti-HBsAg titer between M3/M6 and M4/M7 with days elapsed between M3/M6 and M4/M7 stratified according to age (<40 years and ≥40 years). Spearman correlation test.
