# Supplementary material for: Vaccination Schedule and Age Influence Impaired Responsiveness to Hepatitis B Vaccination: A Randomized Trial in Central Asia
Source: Pathogens. 2024 Dec 9;13(12):1082. doi: 10.3390/pathogens13121082 (PMC11728755; doi:10.3390/pathogens13121082)
Supplement: Supplementary file 1 [file pathogens-13-01082-s001.zip › Table S1.pdf]

Supplementary Table S1. Characteristics of final study population stratified according to age by 10-year increments.

	Age groups	N	0-1-3	0-1-6	*P* value
Participants, N	All	105	53	52	n/a
	<20	5	4	1	n/a
	20-39	25	11	14	n/a
	30-39	21	11	10	n/a
	40-49	27	15	12	n/a
	50-59	20	11	9	n/a
	≥60	7	1	6	n/a
Median age in years (range)	All	40 (17-66)	40 (17-61)	41.5 (19-66)	0.3769 [a]
	<20	18 (17-19)	18 (17-19)	n/a	n/a
	20-29	26 (21-29)	25 (21-29)	26 (22-29)	0.5086 [a]
	30-39	35 (30-39)	35 (30-37)	36 (31-39)	0.4971 [a]
	40-49	45 (40-49)	45 (40-49)	46 (41-49)	0.4156 [a]
	50-59	56 (50-59)	55 (51-59)	56 (53-59)	0.9801 [a]
	≥60	61 (60-66)	n/a	61 (60-66)	n/a
Female sex, n (%)	All	68 (64.8%)	36 (67.9%)	32 (61.5%)	0.4934 [b]
	<20	4 (3.8%)	4 (7.5%)	0 (0%)	0.0434 [b]
	20-29	13 (12.4%)	7 (13.2%)	6 (11.5%)	0.7952 [b]
	30-39	11 (10.5%)	7 (13.2%)	4 (7.7%)	0.3562 [b]
	40-49	19 (18.1%)	9 (17.0%)	10 (19.2%)	0.7647 [b]
	50-59	16 (15.2%)	9 (17.0%)	7 (13.5%)	0.6159 [b]
	≥60	5 (4.8%)	0 (0%)	5 (9.6%)	0.0207 [b]
Median time between 3rd dose and 4th blood draw in days (range)	All	190 (19-275)	214 (25-275)	185.5 (19-249)	0.0105 [a]
	<20	60 (33-217)	47 (33-79)	n/a	n/a
	20-29	145 (28-258)	214 (52-258)	47 (28-204)	0.0026 [a]
	30-39	210 (19-266)	224 (31-266)	195.5 (19-229)	0.2160 [a]
	40-49	193 (25-275)	200 (25-275)	168.5 (28-217)	0.6566 [a]
	50-59	189.5 (25-259)	243 (28-259)	186 (25-210)	0.1460 [a]
	≥60	190 (28-256)	n/a	190 (28-249)	n/a

[a] Mann-Whitney or [b] Chi-squared test was applied for differences between the two vaccination schedules. n/a = not applicable.
