# Supplementary material for: Vaccination Schedule and Age Influence Impaired Responsiveness to Hepatitis B Vaccination: A Randomized Trial in Central Asia
Source: Pathogens. 2024 Dec 9;13(12):1082. doi: 10.3390/pathogens13121082 (PMC11728755; doi:10.3390/pathogens13121082)
Supplement: Supplementary file 1 [file pathogens-13-01082-s001.zip › Table S2.pdf]

Supplementary Table S2. Final study population stratified by education level.

Age groups	All	<20	20-29	30-39	40-49	50-59	≥60
Participants, N	90	5	21	17	21	19	7
No education	1	1	0	0	0	0	0
Primary school	0	0	0	0	0	0	0
Secondary school	10	1	2	2	4	0	1
Vocational education	21	1	2	2	7	5	4
Uncompleted higher education (university)	4	2	2	0	0	0	0
Higher education (university)	54	0	15	13	10	14	2
