# Supplementary material for: Vaccination Schedule and Age Influence Impaired Responsiveness to Hepatitis B Vaccination: A Randomized Trial in Central Asia
Source: Pathogens. 2024 Dec 9;13(12):1082. doi: 10.3390/pathogens13121082 (PMC11728755; doi:10.3390/pathogens13121082)
Supplement: Supplementary file 1 [file pathogens-13-01082-s001.zip › Table S3.pdf]

Supplementary Table S3. Comparison of education level between participants age <40 years and ≥40 years.

	**Age <40**	**Age ≥40**	**P value [a]**
Participants, N			
No education	1/43 (2%)	0/47 (0%)	0.4778
Primary school	0/43 (0%)	0/47 (0%)	>0.9999
Secondary school	5/43 (12%)	5/47 (11%)	>0.9999
Vocational education	5/43 (12%)	16/47 (34%)	0.0138
Uncompleted higher education (university)	4/43 (9%)	0/47 (0%)	0.0483
Higher education (university)	28/43 (65%)	26/47 (55%)	0.3932

[a] Mann-Whitney test was applied for between-age comparisons.
