# Supplementary material for: Vaccination Schedule and Age Influence Impaired Responsiveness to Hepatitis B Vaccination: A Randomized Trial in Central Asia
Source: Pathogens. 2024 Dec 9;13(12):1082. doi: 10.3390/pathogens13121082 (PMC11728755; doi:10.3390/pathogens13121082)
Supplement: Supplementary file 1 [file pathogens-13-01082-s001.zip › Table S4.pdf]

Supplementary Table S4. Anti-HBsAg GMTs after 1st, 2nd, and 3rd vaccine dose, stratified according to age (95% CI).

Age group	M1	M3/M6	M4/M7	*P* value [a]
<20	20.13<br>(0.33, 1225)	121.90<br>(44.10, 337.20)	126.90<br>(34.55, 466.10)	0.3673
20-29	0.13<br>(0.03, 0.49)	8.15<br>(2.59, 25.67)	67.31<br>(34.39, 131.8)	<0.0001
30-39	0.06<br>(0.01, 0.27)	11.05<br>(2.92, 41.75)	36.70<br>(16.47, 81.76)	<0.0001
40-49	0.16<br>(0.05, 0.52)	1.38<br>(0.35, 5.47)	16.74<br>(6.07, 46.18)	<0.0001
50-59	0.47<br>(0.18, 1.21)	0.57<br>(0.11, 2.91)	16.60<br>(3.30, 83.57)	<0.0001
≥60	1.19<br>(0.15, 9.40)	0.16<br>(0.004, 5.50)	5.3<br>(0.09, 311.60)	0.5788

[a] Friedman test across all time points within one age group.
