# Supplementary material for: Vaccination Schedule and Age Influence Impaired Responsiveness to Hepatitis B Vaccination: A Randomized Trial in Central Asia
Source: Pathogens. 2024 Dec 9;13(12):1082. doi: 10.3390/pathogens13121082 (PMC11728755; doi:10.3390/pathogens13121082)
Supplement: Supplementary file 1 [file pathogens-13-01082-s001.zip › Table S5.pdf]

Supplementary Table S5. Anti-HBsAg GMTs of vaccinees after 3rd vaccination stratified according to education level. Data are presented as GMTs with 95% CI.

	**Age <40**	**Age ≥40**	**P value [a]**
Anti-HBsAg GMTs (95% CI)			
No education	n/a	n/a	n/a
Primary school	n/a	n/a	n/a
Secondary school	21.66 (1.95, 240.7)	22.97 (0.92, 570.7)	0.8413
Vocational education	53.51 (10.79, 265.4)	5.96 (0.66, 53.41)	0.3426
Uncompleted higher education (university)	130.7 (18.86, 915.4)	n/a	n/a
Higher education (university)	56.81 (27.31, 118.2)	26,59 (8.88, 79.64)	0.2573

[a] Mann-Whitney test was applied for between-age comparisons.
n/a = not applicable
